# Correction: Intracellular growth of Mycobacterium tuberculosis after macrophage cell death leads to serial killing of host cells

**DOI:** 10.7554/eLife.28205

**Published:** 2017-05-05

**Authors:** Deeqa Mahamed, Mikael Boulle, Yashica Ganga, Chanelle Mc Arthur, Steven Skroch, Lance Oom, Oana Catinas, Kelly Pillay, Myshnee Naicker, Sanisha Rampersad, Colisile Mathonsi, Jessica Hunter, Emily B Wong, Moosa Suleman, Gopalkrishna Sreejit, Alexander S Pym, Gila Lustig, Alex Sigal

Mahamed D, Boulle M, Ganga Y, Mc Arthur C, Skroch S, Oom L, Catinas O, Pillay K, Naicker M, Rampersad S, Mathonsi C, Hunter J, Wong EB, Suleman M, Sreejit G, Pym AS, Lustig G, Sigal A. 2017. Intracellular growth of Mycobacterium tuberculosis after macrophage cell death leads to serial killing of host cells. *eLife* **6**:e22028. doi: 10.7554/eLife.22028.

Published 28, January 2017

Dr Emily B Wong and Dr Moosa Suleman were accidentally left out as co-authors on the work in the originally published version of the article. Their contribution was obtaining and transferring to us bronchoalveolar lavage clinical samples that contained alveolar macrophages. These cells were utilized in Figure 7D, blue line. The corrected list of authors and their affiliations is below:

Deeqa Mahamed^1,2^, Mikael Boulle^1,2,3^, Yashica Ganga^1^, Chanelle Mc Arthur^1,2^, Steven Skroch^1,2^, Lance Oom^1,2^, Oana Catinas^1^, Kelly Pillay^1,2^, Myshnee Naicker^1^, Sanisha Rampersad^1,2^, Colisile Mathonsi^1,2^, Jessica Hunter^1,2^, Emily B Wong^1,4^, Moosa Suleman^5,6^, Gopalkrishna Sreejit^1^, Alexander S Pym^1^, Gila Lustig^1^, Alex Sigal^1,2,3^*

^1^KwaZulu-Natal Research Institute for TB-HIV, Durban, South Africa; ^2^University of KwaZulu-Natal, Durban, South Africa; ^3^Max Planck Institute for Infection Biology, Berlin, Germany; ^4^Division of Infectious Diseases, Massachusetts General Hospital, Boston, United States; ^5^Department of Pulmonology and Critical Care, Nelson R Mandela School of Medicine, University of KwaZulu-Natal, Durban, South Africa; ^6^Department of Pulmonology, Inkosi Albert Luthuli Central Hospital, Durban, South Africa

*Corresponding author

Additional information for Dr Emily B Wong:

Funding:

Burroughs-Wellcome Fund / American Society of Tropical Medicine and Hygiene fellowship

National Institutes of Health K08 AI118538

In addition, we accidentally omitted that Kelly Pillay was funded by a Sub-Saharan African Network for TB/HIV Research Excellence (SANTHE) fellowship.

The article has been corrected accordingly.

